# Laser Excision of Buccal Mucosal Growth: A Case Report

**DOI:** 10.7759/cureus.70180

**Published:** 2024-09-25

**Authors:** Pranjali Gatlewar, Ruchita T Patil, Prasad Dhadse, Shrishti S Salian, Sanehi D Punse

**Affiliations:** 1 Dentistry, Sharad Pawar Dental College and Hospital, Datta Meghe Institute of Higher Education and Research, Wardha, IND; 2 Periodontics and Implantology, Sharad Pawar Dental College and Hospital, Datta Meghe Institute of Higher Education and Research, Wardha, IND

**Keywords:** buccal mucosa, cheek biting, excision, laser, overgrowth

## Abstract

The mucosal tissues are prone to overgrowths as hyperplasia or hypertrophy, which present a spectrum of clinical challenges. These lesions are located at different sites and differ in size and pathology related to their origin. They present as solitary nodules of pink or red on the tongue, gingiva, buccal mucosa and other oral tissues. The most common methods for eliminating overgrowths are scalpel excision, electrosurgery, and laser surgery. Among them, lasers are beneficial to every patient as they do not require anesthetic shots. Even for periodontal surgery, laser surgery is preferred because of its quicker healing, higher rates of re-epithelialization, blood-free surgical site, and superior repair capabilities. A diode laser to remove overgrowth is a rapid, safe treatment with little discomfort or issues after the procedure. This case report describes how a laser is used to excise the mucosal growth with minimal postoperative complications.

## Introduction

Localized overgrowths are one of the most frequently encountered lesions in the oral cavity [1]. The benign lesions rarely showing aggressive features are peripheral giant cell granuloma, pyogenic granuloma, irritational fibroma, and peripheral ossifying fibroma. These lesions are usually the result of trauma or long-term inflammation [2]. Histologically, gingival tissue growth is associated with changes in the extracellular matrix, gingival vessels, cell size, and multiplication [3]. Clinically, they are paler in color than the normal tissue around them. Surface ulceration brought on by subsequent trauma or hyperkeratosis frequently causes the surface to appear white [4].

Commonly occurring as a benign exophytic oral lesion, traumatic fibroma, also called irritant fibroma, arises as a result of tissue injury [5,6]. It results from a prolonged healing process, leading to the formation of granulation tissue and scar, causing the formation of submucosal fibrous mass [7]. Recurrence is rare and can occur due to recurrent trauma to the same area. This lesion does not metastasize [8]. The sites where traumatic fibroma occurs mostly are the tongue, buccal mucosa, and lower labial mucosa [9]. Excision along with its base is the treatment of choice; however, the source of trauma and irritation has to be removed [10].

Lasers are integrated into the dental armamentarium for removing gingival and mucosal overgrowths [11,12]. When used in conservative methods, diode laser therapy exclusively results in favorable outcomes for the treatment of inflamed periodontal tissues [13]. A solid-state semiconductor diode laser was first used in dentistry in 1999. It is composed of gallium (Ga), arsenide (Ar), and other elements like aluminium (Al) and indium (In). Its wavelength falls between 810 and 980 nm. It is a great hemostatic agent because pigments in the soft tissues absorb this energy level [14]. Soft-tissue tuberosity reductions, gingivectomies, gingivoplasties, biopsies, ablation, and some crown-lengthening procedures can all be performed with laser surgery [15]. This case study shows how a diode laser was used to excise a mucosal overgrowth present on the left buccal mucosa.

## Case presentation

**Figure 1:**
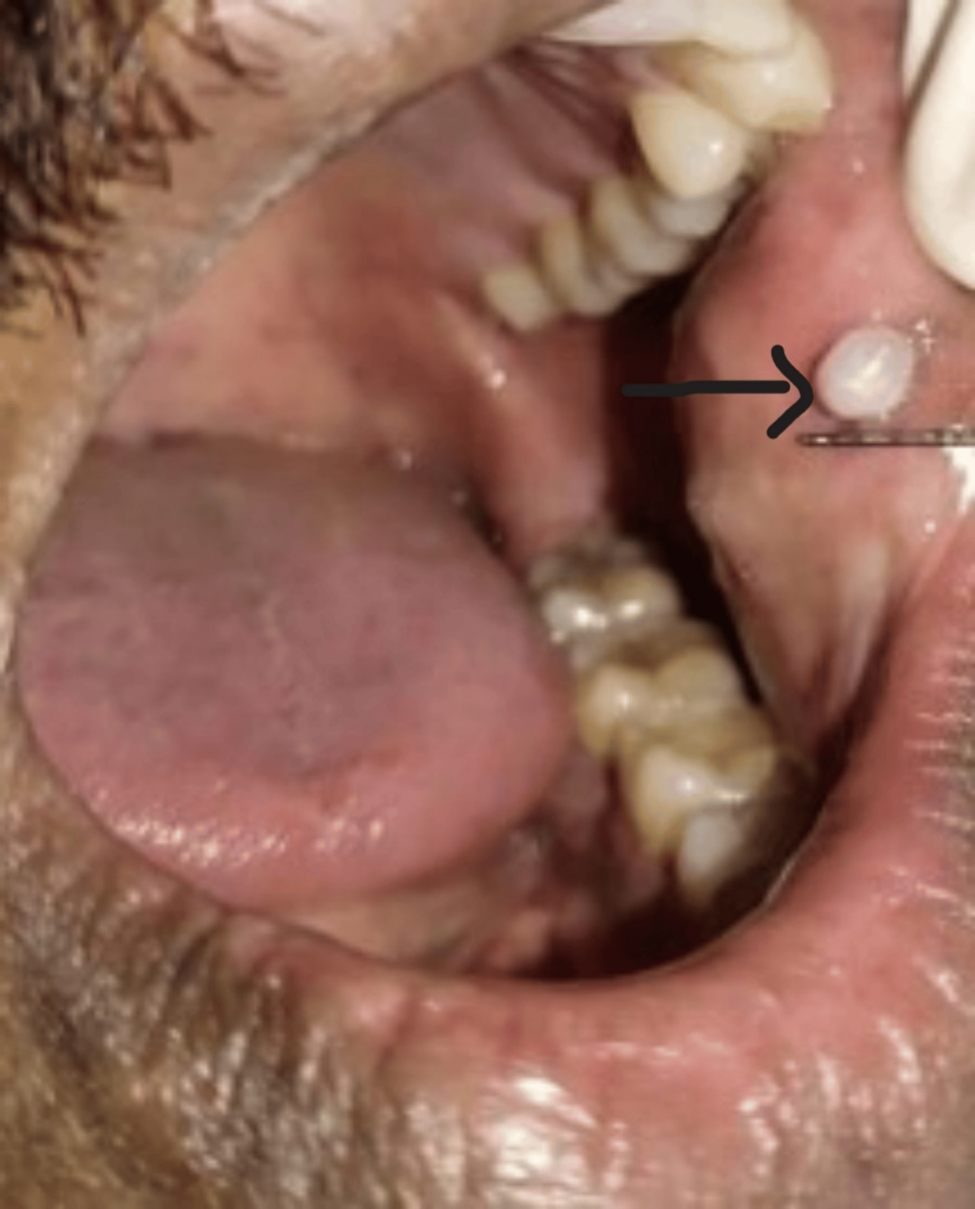
Preoperative photograph: intraoral overgrowth in the left buccal mucosa

A male patient, 42 years old, reported to the Department of Periodontics and Implantology, with a complaint of discomfort caused by a painless intraoral overgrowth, round to oval in shape with a smooth and firm texture, that had been present for three months in the left buccal mucosa as shown in Figure 1. There was no noteworthy medical or dental history. He also complained of recurrent cheek-biting on the same side.

Upon intraoral inspection, an exophytic lesion of approximately 3 x 3 mm was seen on the left buccal mucosa. It was of firm consistency and whitish-pink in appearance. Investigations including bleeding time, clotting time, hemoglobin, and blood glucose level were done in the Department of Oral Pathology as shown in Table 1.

**Table 1:** Hematological findings RBS: Random blood sugar

Investigations	Obtained value	Normal value
Hemoglobin	12.2 g/dL	Males: 12-15.5 g/dL; Females: 11-14.5 g/dL
Bleeding time	1 min 44 sec	1-3 min
Clotting time	3 min 24 sec	1-5 min
RBS	100 mg/dL	Males: 70-160 mg/dL; Females: 70-160 mg/dL

**Figure 2:**
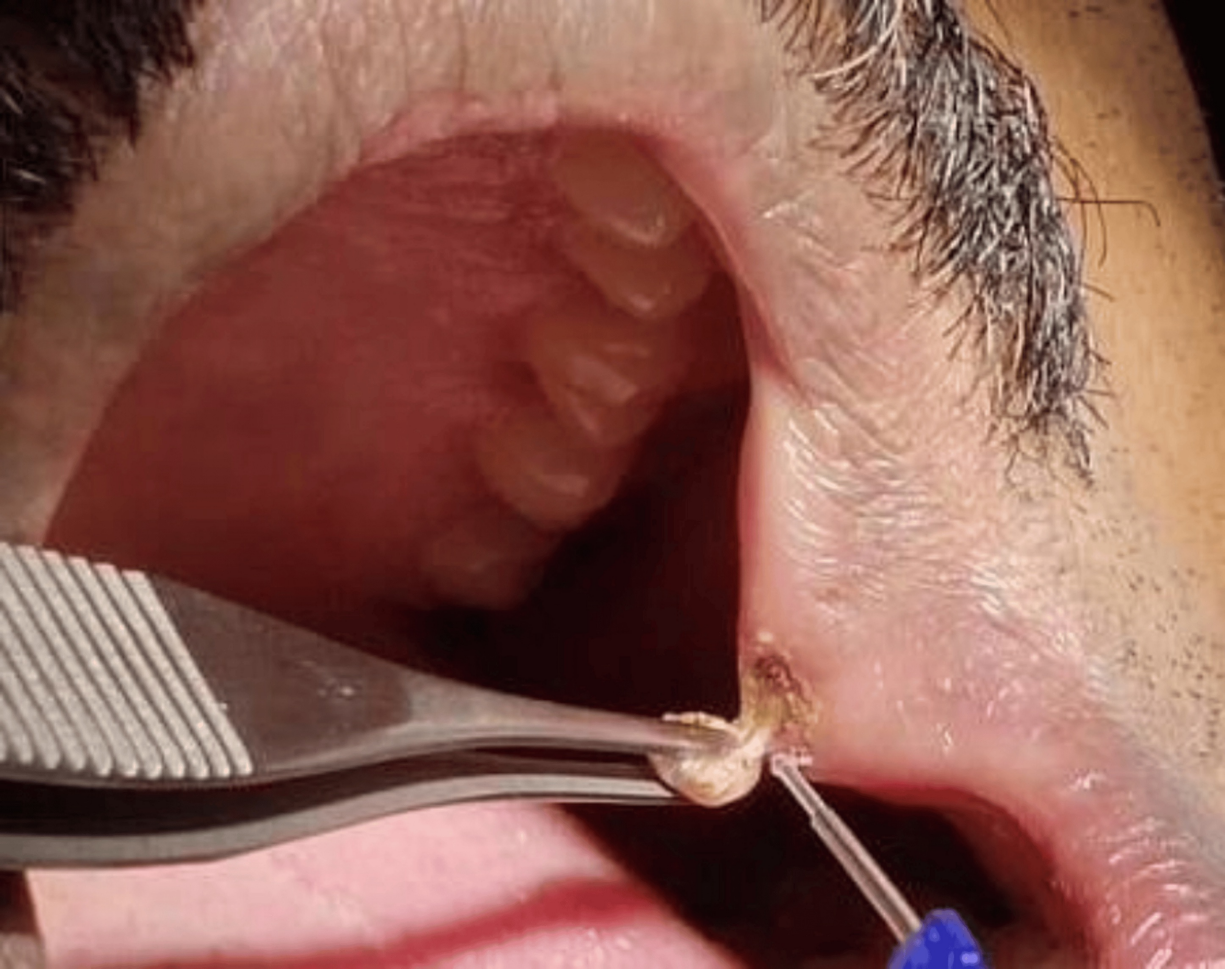
Excision of the mucosal mass using laser

**Figure 3:**
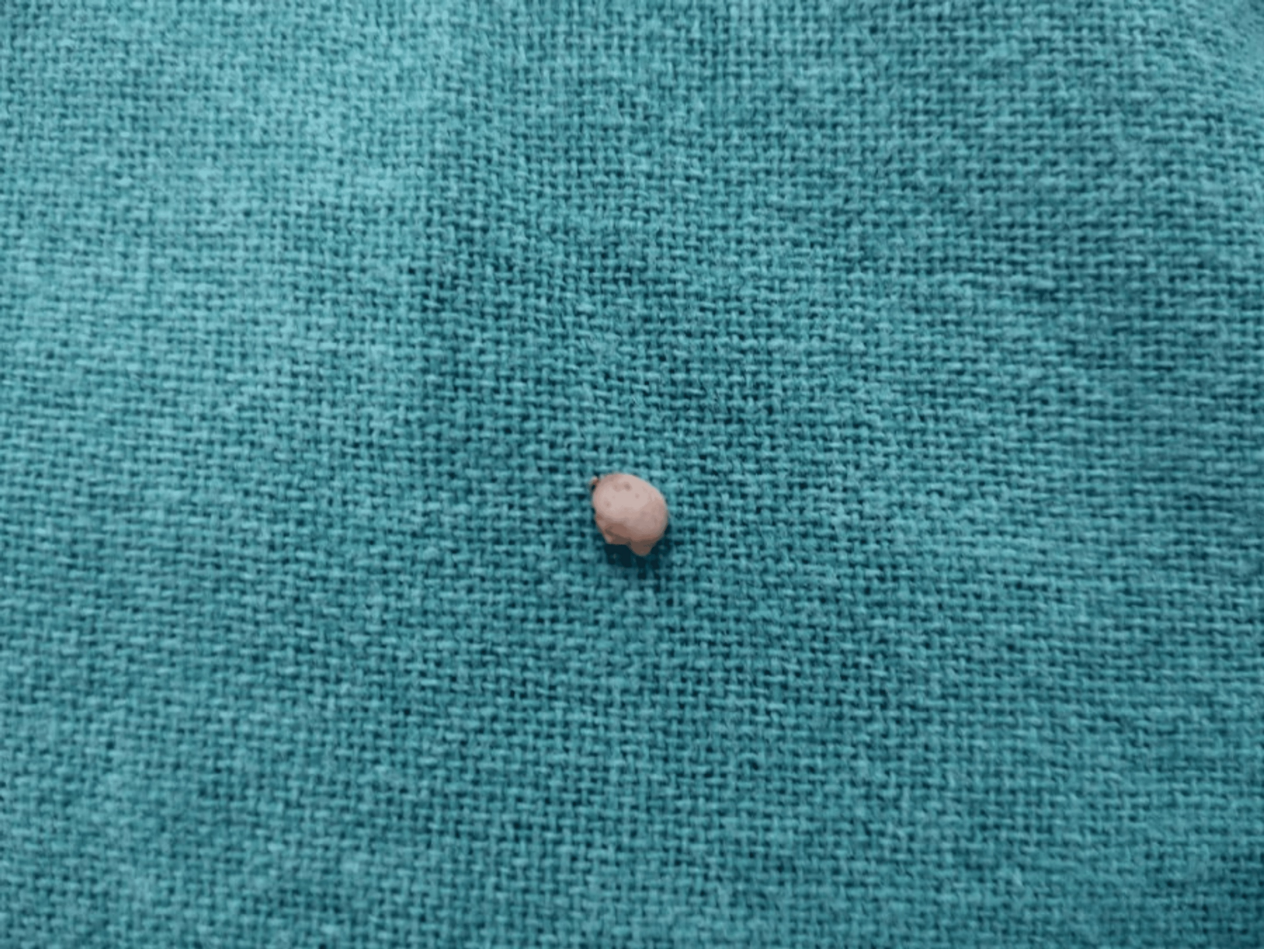
The excised tissue

**Figure 4:**
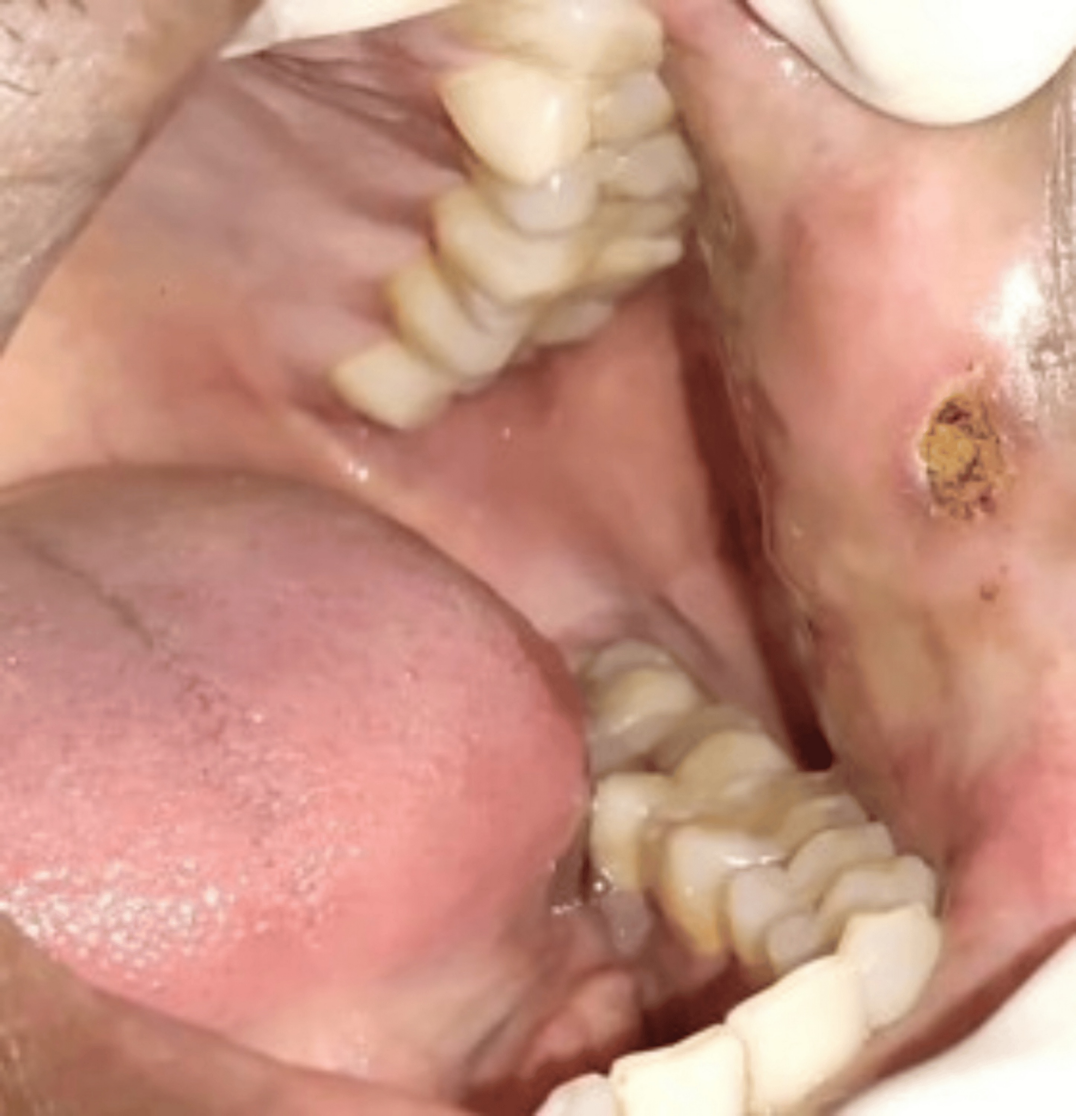
Immediate postoperative photograph after the removal of the mucosal growth

The patient was given an appointment after 10 days for further examination and excision of the mucosal growth using a laser. Before starting the surgery, the patient's informed consent was obtained. To anesthetize the area, a buccal nerve block was given on the left side, and the anesthetic used was 2% lignocaine with adrenaline (1:80,000) concentration. For convenience, the growth was pulled to one side and held in place with non-toothed forceps as shown in Figure 2. The lesion was subsequently fully excised by applying the laser tip to its base as shown in Figure 3. The Biolase® Epic X Diode Laser (Biolase, Foothill Ranch, CA) was used for this procedure. The surgery was carried out more cautiously, with few difficulties and a blood-free operating site as shown in Figure 4.

**Figure 5:**
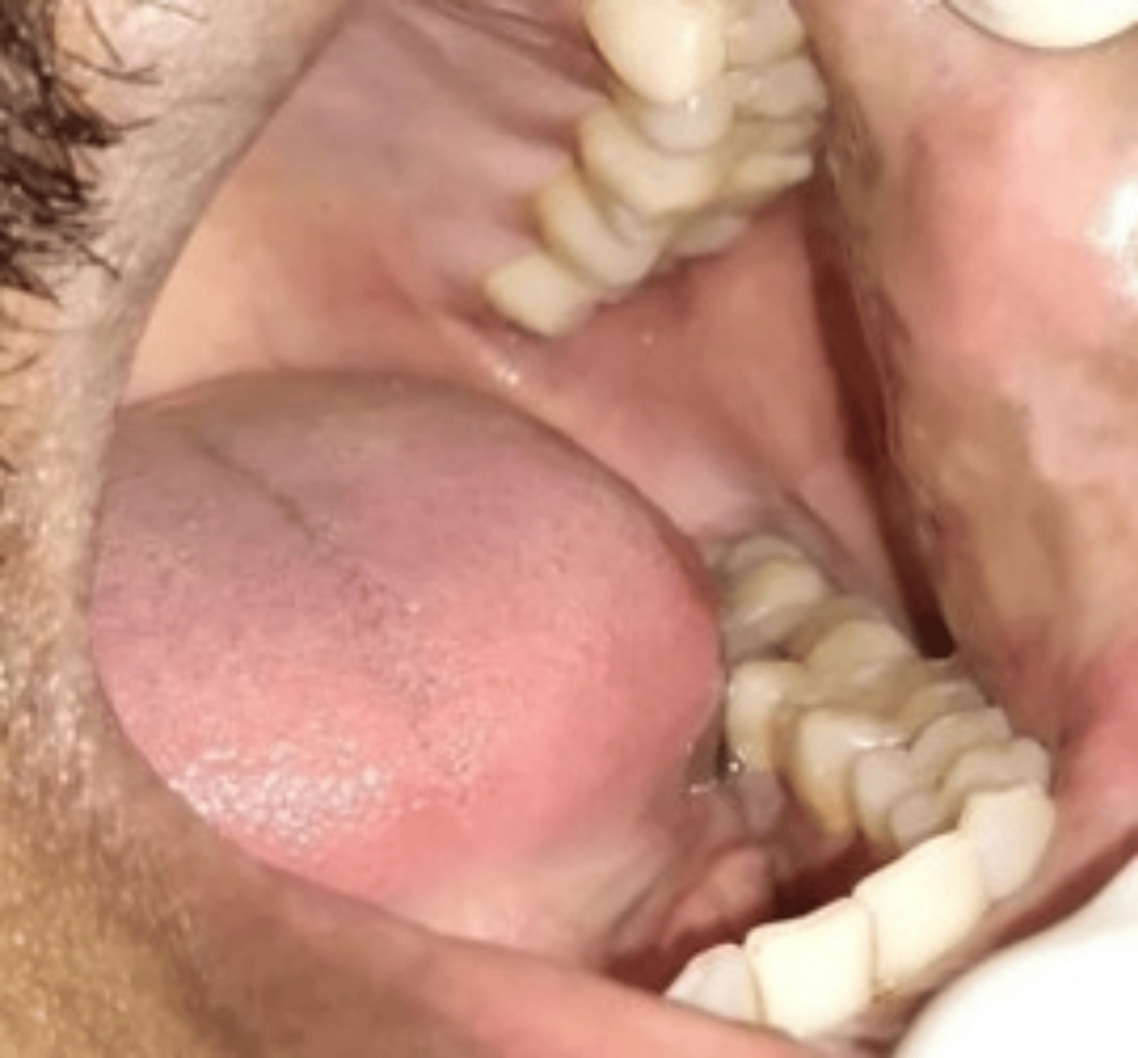
Follow-up after one month showing the healed wound.

A pressure pack was applied to the surgical site to aid in healing after the lesion was completely removed. The patient was given postoperative instructions, which included refraining from eating hot meals and scrubbing the operated side vigorously. Medications were prescribed to the patient including antibiotic amoxicillin (500mg), aceclofenac (100mg), serratopeptidase (15mg), and paracetamol (325mg) for three days. After one month, the patient was recalled for evaluating the operated site. On examination, the wound was found to be healed completely as shown in Figure 5.

## Discussion

It has been noted that most of the localized overgrowths in the oral cavity are likely to be reactive [16]. Other treatment options for such lesions include electrosurgery, laser surgery, and scalpel excision [17]. Complications from the conventional surgery include pre- and post-operative bleeding, wound healing delay, deep anesthesia, edema, scarring, and postoperative pain [17,18].

Oral lesions are more efficiently treated by diode laser radiation, which offers a great, simple, and safe therapy. Some of the advantages of laser surgery over traditional surgical methods include minimal surrounding tissue damage, improved visualization with a bloodless field, reduced post-operative discomfort and recovery time as well as enhanced precision in soft-tissue lesion excision, resulting in decreased formation of scar tissue while preserving the elasticity properties inherent to tumor-bearing tissues [19]. Diode laser causes the biological tissues to become ablated or decomposed (i.e., photophysical effects), and as it works thermomechanically, there is minimal damage to surrounding tissues. Hence such parameters define the need for selective laser surgery. This device has been authorized to treat several types of soft tissue disorders, including but not limited to ablative excision, pocket debridement, incision and drainage, and curettage of the skin [20].

Dentists and other specialists find diode lasers attractive for a variety of reasons: their small size, portability, and low cost compared to high-tech laser equipment. This makes the device available for operation at three different wavelengths: 810, 940, and 980 nm. Carbonization zones showed little heat effects on histological tissue in the low range during an earlier study comparing two laser diode source emissions at 830 and 940 nm [21].

This case study illustrates how overgrowth can be eliminated by diode laser while keeping blood loss minimal, saving time to recover completely. The procedure was painless throughout and no stitches were necessary. Following the excision of the lesion, hemostasis was soon achieved. A three-day antibiotic regimen consisting of aceclofenac (100 mg) + serratopeptidase (15mg) + paracetamol tablet (325 mg) was prescribed in the postoperative period. A follow-up after one month showed that the patient's wound had healed with no evidence of scarring or any other complications. Therefore, diode lasers have evident merits in oral soft tissue surgeries.

## Conclusions

In general, laser-assisted treatment is a promising approach that offers better clinical results and increased patient satisfaction for the treatment of buccal mucosal overgrowths. Lasers offer targeted treatment with less chance of damaging adjacent tissues and faster recovery than traditional surgical approaches because of their precision and low invasiveness. Laser therapy can also improve esthetic results and reduce scarring. When choosing this course of action, it is imperative to take into account the unique elements pertaining to each patient as well as the particular features of the overgrowths.
